# A system dynamics model for national drug policy

**DOI:** 10.1186/2008-2231-22-34

**Published:** 2014-04-01

**Authors:** Akbar Abdollahiasl, Abbas Kebriaeezadeh, Rassoul Dinarvand, Mohammad Abdollahi, Abdol Majid Cheraghali, Mona Jaberidoost, Shekoufeh Nikfar

**Affiliations:** 1 Department of Pharmacoeconomics and Pharmaceutical administration, Pharmaceutical policy research center and Faculty of Pharmacy, Tehran University of Medical Sciences (TUMS), Tehran, Iran; 2 Department of Toxicology and Pharmacology, Faculty of Pharmacy and Pharmaceutical Sciences Research Center, TUMS, Tehran, Iran; 3 Department of Pharmaceutics, Faculty of Pharmacy, TUMS, Tehran, Iran; 4 Food and Drug Organization, Ministry of Health and Medical Education, Tehran, Iran; 5 Department of Pharmacology, University of Baqiyatallah Medical Sciences, Tehran, Iran

**Keywords:** National drug policy, System dynamics, Modeling

## Abstract

**Background:**

Data modeling techniques can create a virtual world to analyze decision systems. National drug authorities can use such techniques to take care of their deficiencies in decision making processes. This study was designed to build a system dynamics model to simulate the effects of market mix variables (5 P’s) on the national drug policy (NDP) indicators including availability, affordability, quality, and rationality. This was aimed to investigate how to increase the rationality of decision making, evaluate different alternatives, reduce the costs and identify the system obstacles.

System dynamics is a computer-based approach for analyzing and designing complex systems over time. In this study the cognitive casualty map was developed to make a concept about the system then the stock-flow model was set up based on the market demand and supply concept.

**Results:**

The model demonstrates the interdependencies between the NDP variables through four cognitive maps. Some issues in availability, willingness to pay, rational use and quality of medicines are pointed in the model. The stock-flow diagram shows how the demand for a medicine is formed and how it is responded through NDP objectives. The effects of changing variables on the other NDP variables can be studied after running the stock-flow model.

**Conclusion:**

The model can initiate a fundamental structure for analyzing NDP. The conceptual model made a cognitive map to show many causes’ and effects’ trees and reveals some relations between NDP variables that are usually forgotten in the medicines affairs. The model also provides an opportunity to be expanded with more details on a specific disease for better policy making about medication.

## Background

Everyone has the inevitable right to achieve the high standard health services, thus this is the duty of health policy makers to promote national drug policies (NDP) in line with national health objectives [1,2]. The NDP objectives are defined as making essential quality medicines available in affordable price for rational use. The NDP as a framework of integrated activities is influenced by various factors especially those arisen from inside the government and the decision making systems. The market-mix variables including product, price, promotion, place and people are also added to other complexities and issues that should be taken into account by national drug authorities (NDAs) [3].

The NDP key indicators should be compatible with health system objectives in terms of effectiveness, financial fairness and responsiveness. Monitoring the processes and their results is so essential, however lack of the live key indicators make it difficult to have a clear picture of the consequences of decisions made by NDAs [4,5].

The NDA and different parties in the ministry of health (MOH) have problems in making unified decisions that would result in amelioration or deterioration of NDP indicators. Surely, the drug systems and decision makers have limited resources and technologies to predict and evaluate the consequences of their decisions. Exploring the previous studies shows a retrospective nature of appraising evidences and key performance indicators that influence the decision making processes in the health systems [6]. In fact the consequences of some NDAs’ decisions are appeared when it cannot be compensated. To overcome such a deficit in decision making, the role of simulation systems for solving the problems is reasonable [7,8].

The NDP is a complex system involving many variables; therefore, a system thinking approach is needed to analyze the roles of influencing factors [9]. To enhance the system efficiency and integrating activities, analysis of processes and evaluation the negative/positive effects of key variables must be addressed.

Qualitative and quantitative improvement in health system necessitates NDAs to provide higher quality services but considering government downsizing and budget constraints, there is no opportunity to increase human and capital resources. Therefore, simulation-based systems can facilitate and accelerate the decision process in order to help policy makers.

System dynamic (SD) is a modeling concept that supports decision systems by breaking them into simpler and smaller subsystems. It helps:

– Shortening the decision process

– Increasing the rationality of actions

– Evaluating the different alternatives

– Reducing the costs

– Decreasing the human-derived mistakes

– Increasing reliability and validity

– Providing potentials for sensitivity analysis and repeatability.

SD founded by Jay Forrester is used to analyze the performance of complex systems [10]. It is typically used for models that represent relationships between system variables, rates of change over time and unequivocal feedbacks [11].

A rational relationship between the functions of the NDP core components and market-mixed variables as the main variables of decision making would enhance the outcomes and effectiveness of decisions. To use SD method, it is essential to add some other constant variables and relations to the model.

Although modeling technique is not a new approach in policy making, it is new in pharmaceutical affairs [12,13]. Nowadays there is no such systematic decision module in Iran while NDAs need such a tool to take care of deficiencies in decision making process. There are some negative and positive variables which affect the NDP. Therefore, building a systemic model can identify, analyze and monitor the negative/positive effects of influential factors and at the end reduces the negative effects and improves positive effects which causes the NDP to promote.

Taking the case of Iran pharmaceutical sector into account, we designed this study to analyze the effects of market mix on the NDP indicators. This study was aimed to investigate the NDP components, helps to rationalize activities and decision making, evaluates different alternatives and increases the cost-effectiveness of interventions.

## Method

In fact SD models are crucial and effective tools for focusing on stock variables and the flows between them. Therefore, it seems using SD as a well-adjusted modeling technique is authentic to respond to the requirements of this study [14,15].

The model should dynamically and quantitatively simulate the core components of NDP (availability, affordability, quality and rational use). Furthermore, it should reflect the interactions between the components and the market-mix variables (price, product, place, people, and promotion). The model should also address the key influencing factors for improvement of health policies.

The NDP is composed of four subsystems: availability, affordability, quality, and rationality. The related variables were listed (Table 1) and the model was developed in a deductive basis in three phases:

– Conceptualization: in this phase, the purpose of the model, the main structure, the boundaries of system and subsystems were developed and the results were demonstrated through a casual network or a cognitive map [16-19]. In addition to the articles and documents, an expert panel (including three decision maker in IR FDA, one expert of SD and two pharmacoeconomists) formed to justify the model.

– Stock-flow modeling: the variables are categorized to level, auxiliary and constant. Then the adjusted model and mathematical equations between the variable were developed. For running the model Vensim PLE software were used. This software makes an opportunity to develop and run system dynamics models in educational or proffessional level [10,20].

– Testing and sensitivity analysis; the model was verified and validated to increase the realty of the simulation. There are some testing methods in SD that would explain in result part [21-23].

**Table 1:** The list of variables those used in the models’ subsystems (A: Auxiliary, C: Constant variable)

	**Variable**	**Description**	**Availability**	**Affordability**	**Quality**	**Rationality**
1	Affordability	Affordability	C	A		
2	Availability of domestic products	Availability of domestic products	A	C		
3	Availability of imported products	Availability of imported products	A	C		C
4	Brand Strength Dom.	Brand Strength domestic products				A
5	Brand Strength Imp.	Brand Strength imported products				A
6	Community promotion	Community promotion				A
7	Competition Dom.	Competition domestic products	A		A	
8	Consumption Dom	Consumption domestic products	A	A		
9	Consumption Imp.	Consumption imported products	A	A		
10	Cost of production	Cost of production			A	
11	Demand Dom.	Demand domestic products	A		A	
12	Demand dom/imp	Share of domestic products’ demand	A		A	
13	Demand Imp	Demand imported products	A		A	
14	Diagnosis accouracy	Diagnosis accouracy				A
15	Distributors stock dom.	Distributors stock domestic products	A			
16	Distributors stock Imp	Distributors stock imported products	A			
17	Drug costs Dom.	Average costs of domestic products	A	A		
18	Drug costs Imp.	Average costs of imported products	A	A		
19	Drug Price Dom.	Average price domestic products	C	C	A	A
20	Drug price Imp.	Average price imported products	C	C	A	A
21	Efficacy	Efficacy				A
22	GDP/Capita	GDP per Capita		C		
23	Global density of pharmacies	Average density of pharmacies in the country	A			
24	Good dispensing practice	Good dispensing practice				C
25	Good lableing	Good lableing				C
26	HouseHold costs	HouseHold costs		C		
27	Import	Volume of imported products	A			
28	Importers	Number of importers	A			
29	Income	Gross national income per capita		C		
30	Induced demand	Induced demand	A			A
31	Informed consumer	Informed consumer				A
32	Intractions	Medicinal intractions				A
33	Market saturation	Market saturation	C			
34	No. distributors	Number of distributors	A			
35	No. Known Patients	Number of known patients				A
36	No. pharmacies	Number of pharmacies	A			
37	No. pharmacists	Number of pharmacists	A			
38	No. physicians	Number of physicians				C
39	No. producers	Number of producers	A			
40	OoP/Household cost	OoP/Household cost		A		
41	OoP/Income	OoP/Income		A		
42	OoP/GDP	OoP/GDP		A		
43	Out of pocket	Out of pocket	A	A		
44	Packaging quality	Packaging quality			A	
45	Patients purchase domestic	Patients purchase domestic products	A	A		
46	Patients purchase Imp.	Patients purchase imported products	A	A		
47	Pharmacies purchase dom.	Pharmacies purchase domestic products	A			
48	Pharmacies purchase Imp	Pharmacies purchase imported products	A			
49	Pharmacies stock domestic	Pharmacies stock domestic products	A			
50	Pharmacies stock Imp	Pharmacies stock imported products	A			
51	Physicians’ K.A.P.	Physicians’ Knoledge/Attitude/practice about rationality				A
52	Polypharmacy	Polypharmacy				A
53	Population	Population	C			
54	Prescriber acceptance	Prescriber acceptance				A
55	Prescription	Prescription				A
56	Prescription with injectables	Prescription with injections				A
57	Prescriptions with Ab	Prescriptions with antibiotic				A
58	Producer profit	Producer profit			A	
59	Producers’ stock	Producers’ stock	A			
60	Production	Production	A		A	
61	Promotion Dom.	Promotion on domestic products	A		A	A
62	Promotion Imp.	Promotion on imported products	A		A	A
63	Quality budget Dom.	Budget for quality improvement of domestic products			A	
64	Quality budget Imp.	Budget for quality improvement of imported products			A	
65	Quality Dom.	Quality index of domestic products	C		A	C
66	Quality Imp.	Quality index of imported products	C		A	C
67	R&D budget	R&D budget			A	
68	Rational prescribing	Rational prescribing				A
69	Rational use	Rational use				A
70	Rationality	Rationality	A			A
71	Real demand	Real demand				A
72	Regional density of medical centers	Regional density of medical centers	A			
73	Regional density of pharmacies	Regional density of pharmacies	A			
74	Regulatory power	Regualatory power			C	
75	RX as OTC	dispensing RX products without prescription				A
76	Safety stock	Safety stock	C			
77	Sales costs	Sales costs			A	
78	Sales value Dom.	Sales value of domestic products	A		A	A
79	Sales value Imp.	Sales value Imported of products	A		A	A
80	Saving/OOP	Saving/OOP		A		
81	Self-treatment	Self-treatment				A
82	Side effects	Side effects				A
83	Social information	Social information				A
84	Stock imported	Stock of imported products	A			
85	Total demand	Total demand	A	A		A
86	Treatment	Treatment		A	A	A
87	User stock Dom.	Stock of domestic products in homes	A			
88	User stock Imp.	Stock of imported products in homes	A			
89	Waste & Exp. Dom.	Waste & expired domestic products	A			
90	Waste & Exp. Imp.	Waste & expired imported products	A			
91	Willing to use	Willing to use				A
92	WTP	Willingness to pay	A	A		

## Results

### Study area

The NDA in Iran -under supervision of MOH- oversees and regulates the provision and utilization of medicines through pharmaceutical division of Food and Drug Administration (IR FDA). The demand of medicines is mainly responded through registered products that are supplied by the public and private manufacturers and importers. IR FDA follows the generic approach and tries to protect domestically produced generic medicines in the market. Two-third of the Iran’s 3.5 billion USD market has been supplied by local manufacturers. A half of manufacturers are presented in the stock market and their main stocks holders are the Social Security Investment Company, Melli Bank Investing Company and Alborz Investing Company; the other half of manufacturing companies and the most importers are owned by private sectors. There are tens of distributors that distribute medicines around the country but the top five covers about 80 percent of the market. The price of all medicines is set by the government through the commission of pricing in IR FDA. The official method of pricing is cost-plus for generic medicines and external reference pricing for branded products; although some country-specific factors such as market size, anti-inflation policies, national economics and some political issues are determinants. Clinical services are provided by both public and private sectors but patients pay the same price for medicines in both sectors. The majority of the people are covered for their treatment costs by three main basic health insurers; they cover about 45 percent of health costs. The medication costs for certain illness including AIDS, TB, Malaria, Hemophilia, Thalasemia, transplantation and vaccination are covered totally by the MOH [24]. The survey on access to medicines found that most general medicines are available and affordable for all - the lowest paid workers as indicator- in both public and private sectors [25,26].

### Logical framework of the model

Our suggested SD conceptual model is composed of two subsystems: NDP objectives and market mix variables. NDP is aimed to improve quality of human life mainly by equitable providing affordable quality drugs for patients who rationally need them. Market mix (5 P’s) are components of a market that are aimed by marketing strategies. The interaction between NDP objectives and market mix components shaped the framework of the model.

Health system is too wide and complicated to be modeled completely in a detailed study; the framework of the model determines how deep the model is supposed to study interactions between NDP and market mix. For exploring the interactions among the variables a SD model is proposed which mainly was structured on the demand of medicines.

**Figure 1:**
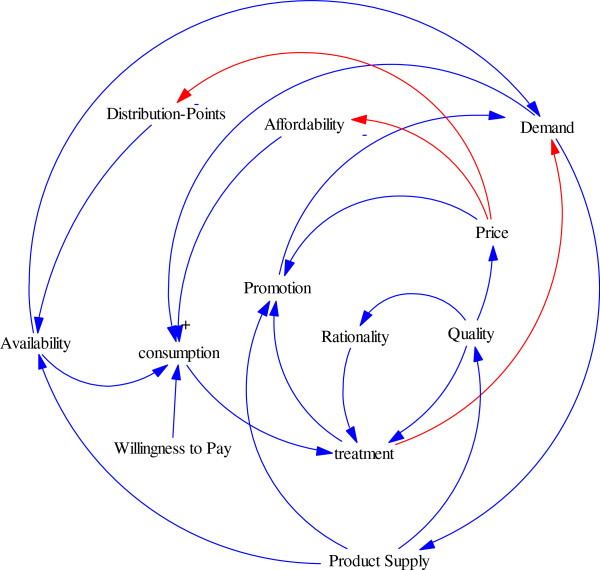
The primary summarized conceptual model for main variables in NDP.

**Figure 2:**
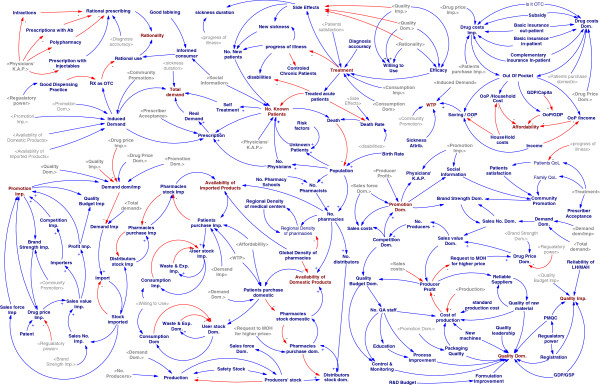
The expanded conceptual model for NDP.

Firstly, a summarized cognitive map of causal loops was described (Figure 1). As mentioned before, the NDP objectives play an important role in helping the policy makers to determine the demand of patients’ medicines. Therefore, the twelve main variables -Affordability, Availability, Consumption, Demand, Distribution-Points, Price, Product Supply, Promotion, Quality, Rationality, Treatment, Willingness to pay (WTP)- formed an overview on the system through the sixteen causal loops. But it was totally obvious that many other variables should be defined to justify the model. All relations in primary structure expanded to a network of variables; to justify the subsystems some other constant or auxiliary variables were added to the model (Figure 2). The expanded model is a casual network that shows the relationships between all variables in NDP. This vast model is for demonstrating the complexity of the system and is essential to break it to smaller parts for detailed analysis.

In Iran, there are two different governmental approaches against imported medicines and domestically produced ones, therefore it was tried to consider these two approaches in studying the main NDP variables. The nature of the model leads to study it in two parts; the conceptual cognitive map was explored in part 1 and quantifying the variables and running their relationships are explained in part 2 in a stock-flow model.

### Part 1–1: Availability

**Figure 3:**
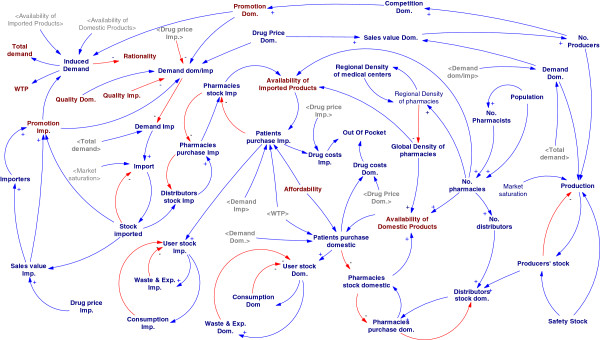
The conceptual model for availability.

According to the logical framework of NDP, the causal diagram of the availability was designed based on two approaches; domestically produced products and imported ones (Figure 3). Availability has been defined as having the essential stock of the product in determined distribution points [1]. Then the number of pharmacies who distribute the product, the distance between them and the level of stock for domestic and imported products determine the level of availability. In the model, both availability variables are placed in two loops that are balanced with patient purchase and pharmacy stock. Pharmacy stock for both domestic and imported product is a part of medicines supply chain which is affected by distributors’ stock and purchase, production, and importation. Patients purchase is influenced by medicines consumption cycles while affordability and WTP are two main variables in these cycles. There is a variable named “demand dom/imp” that shows the ratio of domestically produced product in the market from demand side. This item would balance the availability level of domestically produced medicines versus imported ones.

The other variables which affect the availability loops through the patients purchase are medicines’ stock in patient’s homes and in hospital wards; also the waste and expired medicines are effective.

The other issue in availability is “medical malls” that are places that all medical facilities and physicians’ offices have been concentrated in; based on the current regulations, the number of pharmacies as the dispensing places of medicines is a function of population, distance to other pharmacies and density of medical centers. Medical malls are ideal locations for founding pharmacies but they are against the physical availability of medicines. The IR FDA as the authorized organization for regulating pharmacies allows increasing the number of pharmacies in these regions regardless to the distance to promote the fair income of pharmacies. It makes a reinforced loop to gather more medical firms in such areas and decrease the uniform distribution of pharmacies around the cities; then the level of availability declines.

Availability is not only an essential factor for access to medicines but it can induce the demand in the market. High level of stock which is in favor of availability would increase the financial costs of suppliers then they increase their sales forces whenever they are overstocked; this is one of the causes of the induced demand. Although in the market the data of demand direct the supply, the role of potential market could not be ignored. Potential market that we showed it in the model as “market saturation ratio” is the extra stock of a medicine that should be supplied in addition to real demand for market confidence. “Market saturation” variable that directly related to the safety stock of a medicine in the country, is affecting significantly on other main variable in the model.

### Part 1–2: affordability

**Figure 4:**
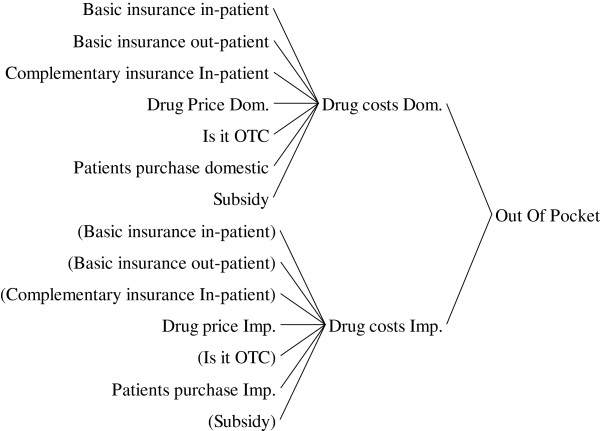
The causes tree for “out of pocket”.

Affordability as having enough money to pay for the medicines has involved many contributors in health system. Out of pocket (OOP) /household costs, OOP/income and OOP/gross domestic products (GDP) per capita are three indicators used to show the affordability of medicines in the model. The coverage of basic and complementary insurances for in-patients and out-patients, the government subsidy on some products such as antihemophilic factors and Iron chelators for Thalassemia, and different pricing approach for over the counter (OTC) medicines are affecting affordability through “out of pocket” (Figure 4).

**Figure 5:**
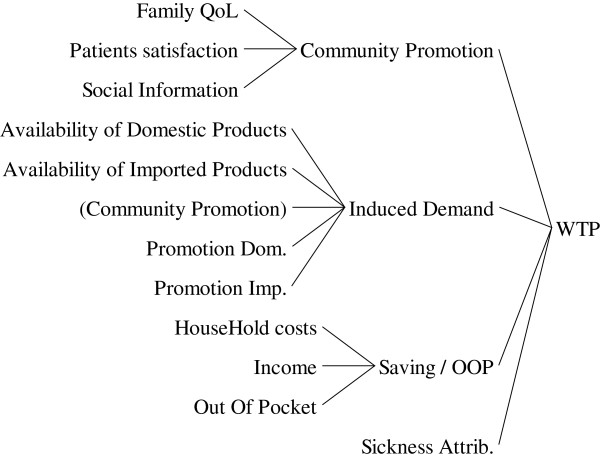
The causes tree for “willingness to pay”.

Although affordability is an important factor to purchase medicines, the role of willingness to pay (WTP) should take into account. Family and social knowledge, promotional activities by suppliers, country and family economical situation, severity of illness and the opportunity costs for medication (the alternative treatments that may exist) tend patients to pay more/less for medicines (Figure 5).

**Figure 6:**
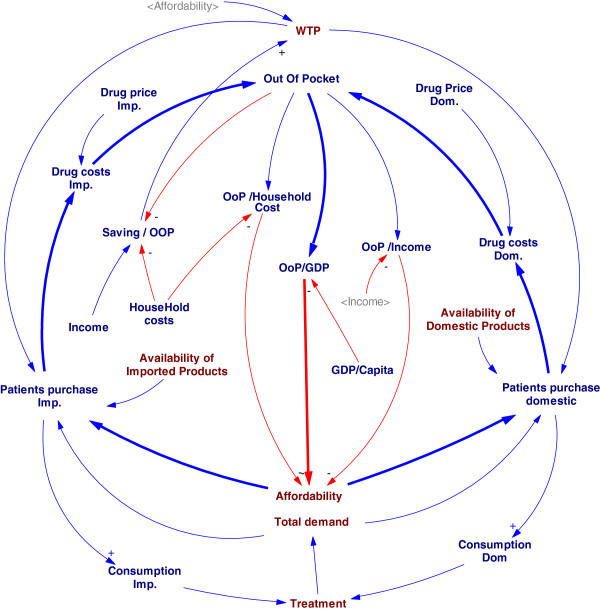
The conceptual model based on affordability.

Despite of different policies against domestic and imported medicines, there are more balanced (negative) than reinforced (positive) loops in this part of the model; all variables that could increase patients’ OOP, would be balanced through reduction of affordability (Figure 6). Prices of domestic and imported drugs that are the most important inputs of affordability loops are the output of suppliers’ requests and negotiation power of the NDA against price increase. The price variables in addition to increase of OOP, can be input of the quality system through sales increase.

### Part 1–3: quality

**Figure 7:**
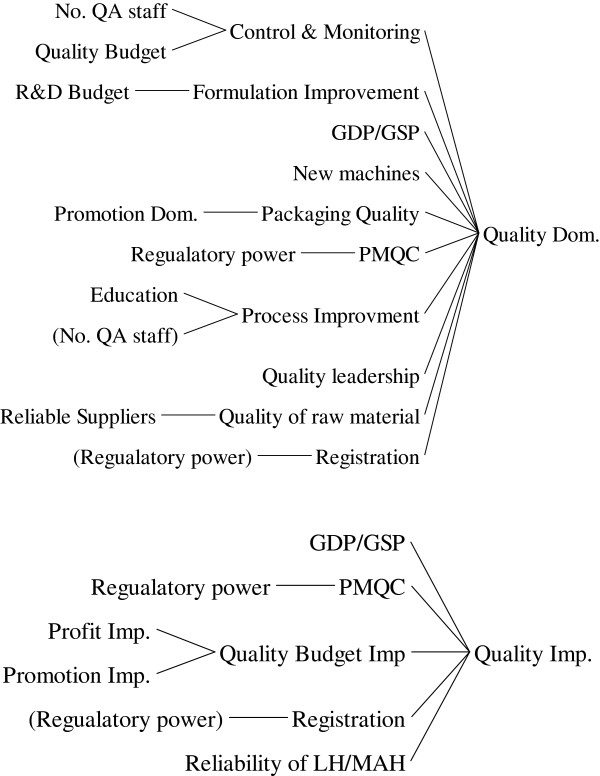
The causes tree for quality.

The quality of medicines not only initiates their safety, efficacy, WTP and patient acceptance but also affects on their share in the market. A wide range of variables influence the quality of domestically produced medicines but a few factors could affect the quality of imported ones (Figure 7). Because NDA has no complete control on the quality of imported medicines in production level in the country of origin, completing registration process and enforcing post marketing quality controls are two main tools for assuring the quality.

**Figure 8:**
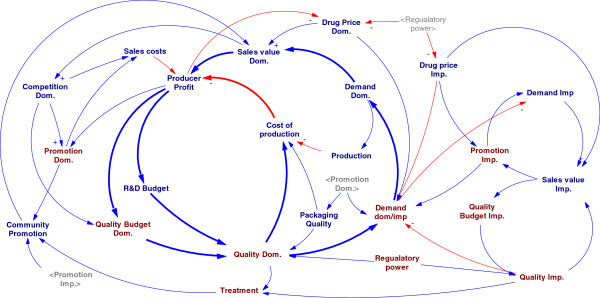
The conceptual model for quality.

The quality of domestic products placed in two feedback loops: the main balanced loop comes from the cost of quality which increases the cost of production and leads to decrease the quality budget due to the profit reduction. The second loop is a reinforced one coming from the increase of demand, sales and market share due to the quality. For imported medicines, there is an only reinforced loop coming from investing on the post manufacturing quality controls and quality promotion (Figure 8).

The role of NDA is crucial in improving quality; NDA can promote the concept of quality management in local pharmaceutical companies, create the opportunity for investing on the quality with rationalizing the prices, regulating and auditing good manufacturing/distributing/storage/laboratory practices in drug supply chain, empowering registration process and post marketing quality control practices.

### Part 1–4: rational use

**Figure 9:**
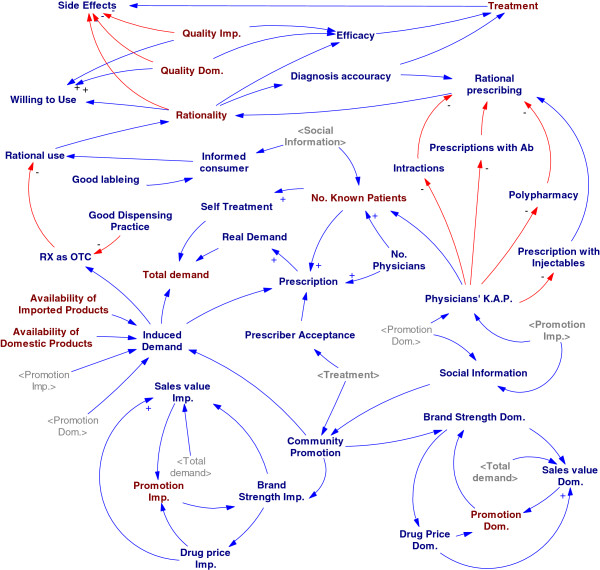
The conceptual model for rationality.

Rational drug use as an important pillar of NDP could clinically, socially, and economically help the health system. In this model rational prescribing, good dispensing practice and giving information to patients are the main determinants of rationality. All promotional and advertising activities not only affect on the public health but also change the demand and subsequently modify activities of supply chains. Sales and promotion reinforce each other in two positive loops (Figure 9). Because there is an information asymmetry in health system, all activities that improve social information about the medicines and change knowledge, attitude and practice of practitioners can positively affect the rational use and prescribing behavior of the medicines.

### Part 1–5: Other important variables

There are some other variables in the model including population, birth and death rate, total demand, responded demand, epidemiological indices, number of physicians and pharmacists and diagnosis accuracy that help to complete system for simulation. Although treatment of patients is the main objective of medication, right diagnose, patient compliance, efficacy and side effects can change the treatment progress. Consuming the medicine is not the end of the treatment chain, many chronic diseases are never cured and patients should consume their medicine forever to control the progress of the disease or improve their quality of lives; thus they always stay on the medicine demand cycle. In spite of the demand for main illness treatment, treating the side effects and new sicknesses have some other negative forces on the treatment cycle and leads to new demands. The patients’ death in chronic diseases and healing in acute ones removes the patients from the treatment cycle and reduce the demand.

### Part 2–1: The stock-flow model

**Figure 10:**
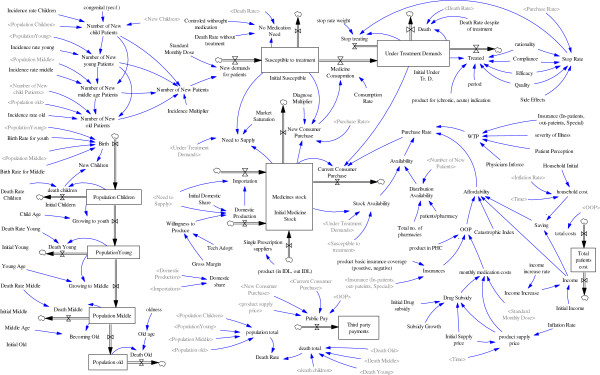
The stock flow model for NDP.

Figure 10 shows the stock flow diagram developed based on the mentioned conceptual casualty network. Population, demand and stock are three bunches of stock variables in the model. Population has divided into four stock variables due to age structure of the country. The incidence rates for each age group, the diagnosis rate and the standard dose of medicine would project the number of susceptible people for treatment that makes the demand. The unit used for demand variables was defined daily dose [27]. Every demand –“susceptible to treat”- that is responded - diagnosed, afforded, provided, purchased and consumed -will move to the variable named “responded demand” (Figure 10). Death and stopping treatment are the exit ways of this stock variable for chronic patients; treating rate is the other exit way for acute ones.

All domestic producers and importers collect their supplied medicine in a stock variable called medicine stock. The level of medicine stock variable is higher than the demand based on market saturation rate. The variable “Medicine stock” has two existence way; all demands that can be responded including new demands and current chronic consumers would reduce the medicine stock through these existence ways called “purchase rates” channels. The purchase rate has made by affordability, availability and WTP.

The quality and rationality related variables put their effects on auxiliary variables called “stop rate” that reduce the number of current consumers.

The variables, their units and the equations were defined on Vensim PLE (academic version) and the model was executed for a 120 months period.

The model was run without any mistakes and the influence of any changes on any variables could be explored on the time trend graphs on other variables that were made by the software.

### Part 3–1: Validity Tests and sensitivity analysis

There are a wide variety of tests for verification and validation of SD models. To assess the structure, dimensional consistency, extreme conditions and robustness of equations under stress situations are used. For testing behavior reproduction the pattern of outputs are compared with real data. Then the model was tested not only for outputs but also for internal structure [23]. Direct structure tests including extreme-conditions and dimensional consistency was done on all major variables by the software (Vensim). Also the expert panel of the study was revising the structure and casualty relations for many times to reach to the optimum situation.

For testing the structure behavior, some major variables including population groups were compared to real data but for some variables we had no real data for comparing and the expert panel tried to justify them.

Because the concept of modeling in NDP is new in Iran and there are a few written documents about it, reaching to a consensus in the expert panel on the result of the model was difficult; it is challengeable for other experts yet. Some extreme and different conditions that the model tested on them were acute versus chronic, high prevalence versus rare disease, cheap versus expensive treatment and rational high quality drugs versus irrational low quality.

Although for testing the validity, a kind of sensitivity analysis was done but for performing sensitivity analysis all rate variables and initial values are changed in a wide range (even wider than real situation) and the behavior of the model and the value of other major variables were studied. Because there are a lot of variables in the model that should be adjusted with a specific disease and its major treatments, the range of the variables’ value significantly depend on the value of other variables. For example if we adjust the model with a rare congenital disease with a full subsidized medicines, availability, affordability and quality variables in the model is no sensitive to the ex-work price or death rate of adults; but it is hugely sensitive to the birth rate.

## Discussion

The model is targeted to help policy makers as a decision support system (DSS) with analyzing interrelationships between availability, affordability, quality and rational use of medicines. The casual network was formed by about 140 selected variables made a crowded cognitive map in the conceptual phase that was too complex to interpret so it forced the model to break into four main subsystems. The challenges developed in defining the borders of the subsystems, caused some intersectional variables to be repeated in more than one subsystem.

The stock-flow model has been set based on the demand and supply concept. This demand was made by the population structure and incidence rates. We had to break the population to four stock variables due to the population structure of Iran. Because of the lack of disease epidemiology data in Iran we used any data from any country for covering incidence rates. It was thought this lack of data could be covered by the ability that is in SD to do a wide range of sensitivity analysis. The supply side was summarized to a few level and auxiliary variables that comes to the model as input variables, then it can be expanded to more detailed models in supplementary studies.

Availability subsystem consists of the supply side of the stock-flow model and number of pharmacies as the constant variable; although increasing the number of pharmacies can improve the availability, it cannot overwhelm the total stock situation; the total stock of the modeled medicine comes from the total demand through domestically production and imports. The number of pharmacies under the control of the government has a slow growth due to low population growth rate.

The insurance system and subsidization that play the main role in affordability subsystem present themselves as two constant variables in the stock-flow diagram. The role of insurance organizations can be explained based on the other variables in health financial system. There is a stock variable in the model that shows the cumulative medication costs of the illness and demonstrates the time when patients could fall in catastrophic expenses. The model shows only cancer and autoimmune patients can fall in the catastrophic expenses; medication for normal high burden diseases including cardiovascular, diabetes, central nervous disorders and gastro intestinal are too cheap to send patients to financial failure.

Quality and rationality in the stock-flow diagram are not in the core of the model; they can just affect the model as a foreign control knob.

It was attempted to use mathematical equations between variables than regression equations. Thus, we had to select some variables that can be adopted with it.

## Conclusion

The model can initiate a fundamental structure for analyzing NDP. The conceptual model made a cognitive map for NDP that not only shows many causes and effects trees but also reveals some relations between NDP variables that are usually forgotten or ignored in the medicines affairs:

– The role of centralized medical centers in reducing the availability of medicines; although the model is silent on the effects of reducing profit of pharmacies on availability [28].

– It had already been demonstrated the increasing share of imported medicines in the market [29] but this study demonstrates the influence of importers’ promotional activities on expanding the market and quality of domestically produced medicines.

– The effects of the patients’ WTP on purchasing their medicines and the demand for the medicine.

– The bigger role of prescriber than consumer in rational use of medicines.

– The mutual effects of overstocking in domestic or imported products on supplying and promotion activities.

– The influence of quality and rational use on the patients’ willingness to use.

There are also some special points in the model that play significant roles in the NDP that should be more notified:

– The amount of medicines that stocked in patients’ homes. It can be the reason that the sales of pharmaceutical usually have no direct relation to health indices [30].

– The effects of medication on the population groups.

– The effect of brand names on the quality.

– The influence of regulatory power on the quality and the supply of medicines that was also explained in other studies [31,32].

Overall this model provides 52 control knobs for the modeler to adjust the model with a selected medicine in a specific disease. Then 121 level and auxiliary variable trends can clarify the consequences of any changes before making any decisions in the NDA.

Linking this model to some real live epidemiological and disease surveillance databases in the country could create a decision support system to help decision making.

The stock-flow model not only shows some relations between NDP variables but provide a framework for other more detailed studies.

## Competing interests

The authors declare that they have no competing interests.

## Authors’ contributions

AA plan the project, set up the panels, developed the primary models, data analysis and drafted the paper, AK and RD conceived and revised the model and supervised the project, MA gave consultation on the study design and edited the draft, AC gave consultation on the conceptual model, SN revised the conceptual model and gave consultation on the medication procedures, MJ revised the stock-flow model and data analysis. All authors read and approved the final manuscript.
